# Quinacrine Inhibits ICAM-1 Transcription by Blocking DNA Binding of the NF-κB Subunit p65 and Sensitizes Human Lung Adenocarcinoma A549 Cells to TNF-α and the Fas Ligand

**DOI:** 10.3390/ijms18122603

**Published:** 2017-12-02

**Authors:** Misuzu Harada, Kyoko Morimoto, Tetsuya Kondo, Reiko Hiramatsu, Yuji Okina, Ryo Muko, Iyo Matsuda, Takao Kataoka

**Affiliations:** 1 Department of Applied Biology, Kyoto Institute of Technology, Matsugasaki, Sakyo-ku, Kyoto 606-8585, Japan; mi-piano.a.sax2@hotmail.co.jp (M.H.); 073128@gmail.com (K.M.); shorthair1994@gmail.com (T.K.); crystal.flower.reiko@gmail.com (R.H.); nakioyou@gmail.com (Y.O.); hosiimo65ryo@hotmail.co.jp (R.M.); citrusiyo.m14325@gmail.com (I.M.); 2 The Center for Advanced Insect Research Promotion (CAIRP), Kyoto Institute of Technology, Matsugasaki, Sakyo-ku, Kyoto 606-8585, Japan

**Keywords:** quinacrine, nuclear factor κB (NF-κB), tumor necrosis-α (TNF-α), intercellular adhesion molecule-1 (ICAM-1), the Fas ligand

## Abstract

Quinacrine has been used for therapeutic drugs in some clinical settings. In the present study, we demonstrated that quinacrine decreased the expression of intercellular adhesion molecule-1 (ICAM-1) induced by tumor necrosis factor (TNF)-α and interleukin-1 (IL-1) α in human lung adenocarcinoma A549 cells. Quinacrine inhibited ICAM-1 mRNA expression and nuclear factor κB (NF-κB)-responsive luciferase reporter activity following a treatment with TNF-α and IL-1α. In the NF-κB signaling pathway, quinacrine did not markedly affect the TNF-α-induced degradation of the inhibitor of NF-κB or the TNF-α-induced phosphorylation of the NF-κB subunit, p65, at Ser-536 and its subsequent translocation to the nucleus. In contrast, a chromatin immunoprecipitation assay showed that quinacrine prevented the binding of p65 to the ICAM-1 promoter following TNF-α stimulation. Moreover, TNF-α and the Fas ligand effectively reduced the viability of A549 cells in the presence of quinacrine only. Quinacrine down-regulated the constitutive and TNF-α-induced expression of c-FLIP and Mcl-1 in A549 cells. These results revealed that quinacrine inhibits ICAM-1 transcription by blocking the DNA binding of p65 and sensitizes A549 cells to TNF-α and the Fas ligand.

## 1. Introduction

Inflammation plays a critical role in different stages of cancer development and progression [1,2]. Nuclear factor κB (NF-κB) is one of the transcription factors responsible for inflammation-promoted cancers [1,2]. The NF-κB signaling pathway is activated by pro-inflammatory cytokines, bacterial components, viruses, and DNA-damaging agents, and induces the expression of various genes that are essential for inflammation, innate immunity, and survival [3]. NF-κB is constitutively activated in many cancer cells, and contributes to proliferation, the prevention of apoptosis, angiogenesis, metastasis, and energy metabolism [4,5]. To date, a number of small-molecule inhibitors that target the NF-κB signaling pathway have been identified [6,7].

Upon stimulation with pro-inflammatory cytokines, such as tumor necrosis factor (TNF)-α and interleukin-1 (IL-1), different sets of adaptor proteins are recruited to their receptors and trigger the activation of the inhibitor of the NF-κB (IκB) kinase complex [8,9,10]. In the classical NF-κB pathway, the IκB kinase complex directly phosphorylates IκBα, which sequesters the NF-κB heterodimer p65 (also known as RelA) and p50 in the cytosol, leading to the ubiquitination and proteasomal degradation of IκBα [8,11]. The p65 and p50 heterodimers subsequently undergo translocation from the cytoplasm to the nucleus, where they bind κB sites and activate a number of target genes [12,13]. p65 is known to undergo post-translational modifications, such as phosphorylation [4,14,15]. Previous studies reported that the phosphorylation of p65 at Ser-536 within the transactivation domain was mediated by a number of protein kinases and stimulated transcriptional activity [4,14,15]. 

Quinacrine (4-*N*-(6-chloro-2-methoxyacridine-9-yl)-1-*N*,1-*N*-diethylpentane-1,4-diamine) is a 9-aminoacridine derivative that has been used as a therapeutic drug for parasite infections and inflammatory diseases [16]. Quinacrine has multiple cellular targets and is regarded as a potential therapeutic agent for cancer [16]. Quinacrine has been shown to inhibit NF-κB-dependent signaling and gene expression in normal cells and cancer cells [17,18,19,20,21,22,23,24]. Moreover, quinacrine sensitizes cancer cells to chemotherapeutic drugs and tumor necrosis factor-related apoptosis-inducing ligand (TRAIL) [20,24,25,26]. However, the mechanisms underlying the inhibition of the NF-κB pathway by quinacrine have not yet been elucidated. In the course of our screening, quinacrine was initially found to inhibit the expression of intercellular adhesion molecule-1 (ICAM-1) in response to TNF-α in human lung adenocarcinoma A549 cells. ICAM-1 expression is mainly up-regulated by NF-κB and regulates immune responses [27,28]. ICAM-1 also plays a role in cancer metastasis [29]. In the present study, we further investigated the inhibitory mechanisms of quinacrine on NF-κB-dependent ICAM-1 expression. Our results revealed that quinacrine inhibited ICAM-1 mRNA expression by blocking the binding of p65 to the ICAM-1 promoter.

## 2. Results

### 2.1. Quinacrine Inhibits the Expression of the Cell-Surface ICAM-1 Protein Induced by Inflammatory Cytokines

**Figure 1:**
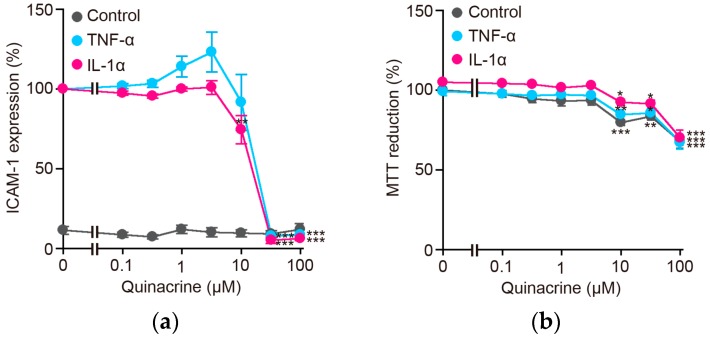
Quinacrine inhibits cell-surface intercellular adhesion molecule-1 (ICAM-1) protein expression induced by TNF-α and IL-1α. (**a**) A549 cells were preincubated with various concentrations of quinacrine for 1 h and then incubated with TNF-α (2.5 ng/mL) or IL-1α (0.25 ng/mL) or without (control) cytokines, in the presence or absence of quinacrine, for 6 h. ICAM-1 expression in TNF-α-stimulated cells and IL-1α-stimulated cells without quinacrine was set to 100%. ICAM-1 expression in unstimulated cells was calculated based on TNF-α-stimulated cells without quinacrine. Data are shown as the mean ± SE of three independent experiments. ** *p* < 0.01 and *** *p* < 0.001 indicate significant differences from the control. (**b**) A549 cells were preincubated with various concentrations of quinacrine for 1 h and then incubated with TNF-α (2.5 ng/mL) or IL-1α (0.25 ng/mL) or without (control) cytokines, in the presence or absence of quinacrine, for 6 h. Cell viability was evaluated by the MTT assay. MTT reduction was calculated based on unstimulated cells without quinacrine as 100%. MTT reduction (%) is shown as the mean ± SE of three independent experiments. * *p* < 0.05, ** *p* < 0.01 and *** *p* < 0.001 indicate significant differences from the control.

Human lung adenocarcinoma A549 cells express cell-surface ICAM-1 when treated with inflammatory cytokines [30,31]. Quinacrine was initially found to reduce TNF-α-induced ICAM-1 expression in A549 cells. A549 cells were preincubated with quinacrine for 1 h and then incubated with TNF-α or IL-1α. A 6-h incubation with TNF-α or IL-1α markedly augmented the amount of cell-surface ICAM-1 protein (Figure 1a). ICAM-1 expression was diminished by quinacrine in a dose-dependent manner and almost completely at 32 to 100 µM in A549 cells (Figure 1a). Under the same conditions, quinacrine partially decreased cell viability (Figure 1b). These results indicate that quinacrine inhibits cell-surface ICAM-1 expression induced by TNF-α or IL-1α.

### 2.2. Quinacrine Inhibits the Expression of ICAM-1 mRNA Induced by Inflammatory Cytokines

**Figure 2:**
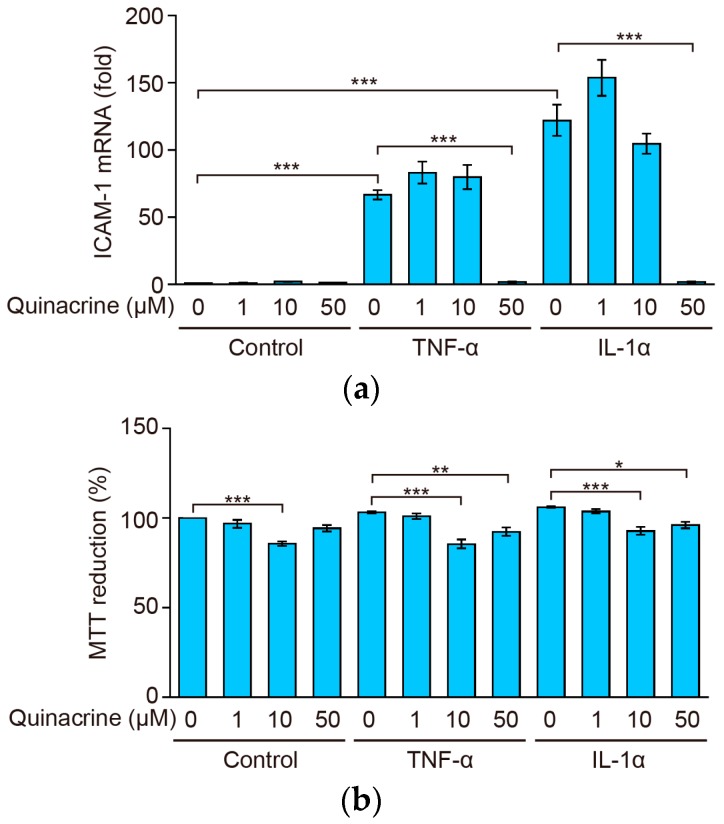
Quinacrine inhibits ICAM-1 mRNA expression induced by tumor necrosis factor (TNF)-α and interleukin 1 (IL-1)α. (**a**) A549 cells were preincubated with various concentrations of quinacrine for 1 h and then incubated with TNF-α (2.5 ng/mL) or IL-1α (0.25 ng/mL) or without (control) cytokines, in the presence or absence of quinacrine, for 2 h. ICAM-1 mRNA expression was analyzed by quantitative-PCR. ICAM-1 mRNA (fold) is shown as the mean ± SE of three independent experiments. *** *p* < 0.001 indicates significant differences from the TNF-α or IL-1α stimulation. (**b**) A549 cells were preincubated with various concentrations of quinacrine for 1 h and then incubated with TNF-α (2.5 ng/mL) or IL-1α (0.25 ng/mL) or without (control) cytokines, in the presence or absence of quinacrine, for 2.5 h. Cell viability was evaluated by the MTT assay. MTT reduction (%) is shown as the mean ± SE of three independent experiments. * *p* < 0.05, ** *p* < 0.01 and *** *p* < 0.001 indicate significant differences from the control.

ICAM-1 is predominantly up-regulated at the transcriptional level [27]. We next investigated the effects of quinacrine on ICAM-1 mRNA. A 2-h stimulation with TNF-α or IL-1α resulted in ICAM-1 mRNA expression that was approximately 70-fold and 120-fold stronger, respectively, than that in unstimulated cells (Figure 2a). A549 cells were preincubated with quinacrine for 1 h and then incubated with TNF-α or IL-1α for 2 h. Quinacrine inhibited ICAM-1 mRNA expression almost completely at 50 µM (Figure 2a). A549 cells were preincubated with quinacrine for 1 h and then incubated with TNF-α or IL-1α for 2.5 h. Quinacrine only slightly, if at all, decreased cell viability at concentrations up to 50 µM for 2.5 h (Figure 2b). These results indicate that quinacrine inhibits ICAM-1 mRNA expression induced by TNF-α or IL-1α, without reducing cell viability.

### 2.3. Quinacrine Inhibits NF-κB-Responsive Luciferase Activity Induced by Inflammatory Cytokines

**Figure 3:**
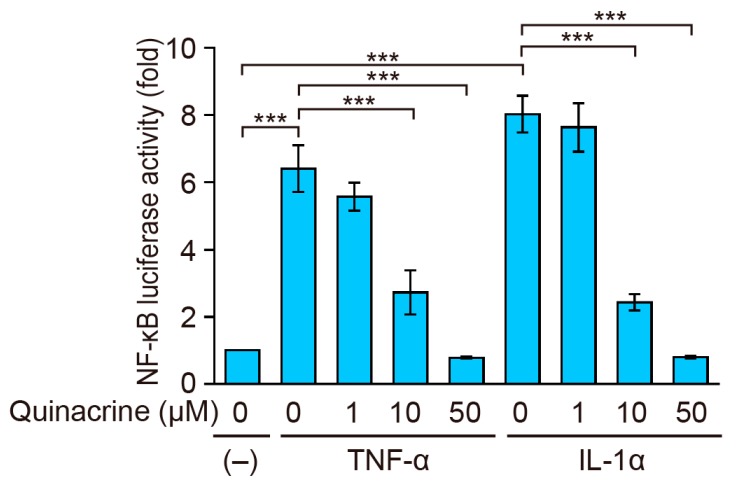
Quinacrine inhibits NF-κB-responsive luciferase activity induced by TNF-α and IL-1α. A549 cells were transiently transfected with the NF-κB-responsive firefly luciferase reporter and the cytomegalovirus promoter-driven *Renilla* luciferase reporter for 24 h. A549 cells were preincubated with various concentrations of quinacrine for 1 h and then incubated with TNF-α (2.5 ng/mL) or IL-1α (0.25 ng/mL) or without cytokines (–), in the presence or absence of quinacrine, for 2.5 h. Luciferase activity (fold) is shown as the mean ± SE of three independent experiments. *** *p* < 0.001 indicates significant differences from the TNF-α or IL-1α stimulation without quinacrine.

In order to investigate the effects of quinacrine on NF-κB-dependent gene expression, the NF-κB reporter assay was performed. A549 cells were preincubated with quinacrine for 1 h and then incubated with TNF-α or IL-1α for 2.5 h. A stimulation with TNF-α or IL-1α induced approximately 6-fold and 8-fold increases, respectively, in NF-κB-responsive luciferase reporter activity, in A549 cells (Figure 3). TNF-α- and IL-1α-induced NF-κB-dependent luciferase activities were reduced by quinacrine at concentrations of more than 10 µM and almost completely at 50 µM (Figure 3). These results indicate that quinacrine inhibits the NF-κB-responsive luciferase reporter activity.

### 2.4. Quinacrine Inhibits TNF-α-Induced ICAM-1 Protein Expression and NF-κB-Responsive Luciferase Activity in Other Cancer Cell Lines

**Figure 4:**
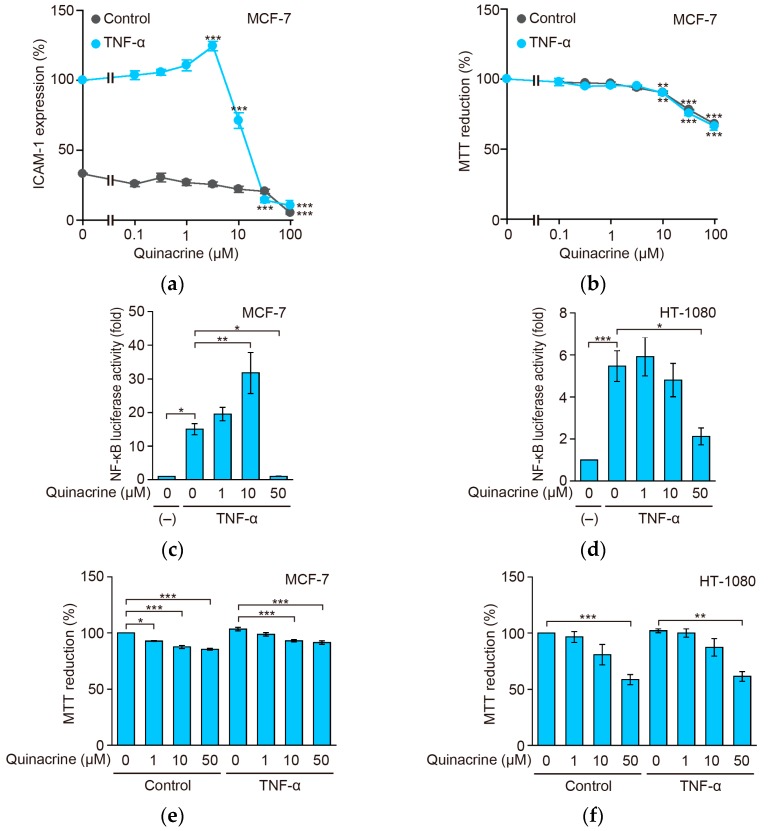
Quinacrine inhibits TNF-α-induced ICAM-1 expression and NF-κB-responsive luciferase activity in other cancer cell lines. (**a**) MCF-7 cells were preincubated with various concentrations of quinacrine for 1 h and then incubated with TNF-α (2.5 ng/mL) or without (control) cytokines, in the presence or absence of quinacrine, for 6 h. ICAM-1 expression in TNF-α-stimulated cells without quinacrine was set to 100%. Data are shown as the mean ± SE of three independent experiments. *** *p* < 0.001 indicates significant differences from the control. (**b**) MCF-7 cells were preincubated with various concentrations of quinacrine for 1 h and then incubated with TNF-α (2.5 ng/mL) or without (control) cytokines, in the presence or absence of quinacrine, for 6 h. Cell viability was evaluated by the MTT assay. MTT reductions were calculated based on unstimulated cells without quinacrine as 100%. MTT reduction (%) is shown as the means ± SE of three independent experiments. ** *p* < 0.01 and *** *p* < 0.001 indicate significantly differences from the control. (**c**,**d**) MCF-7 cells (**c**) and HT-1080 cells (**d**) were transiently transfected with the NF-κB-responsive firefly luciferase reporter and cytomegalovirus promoter-driven *Renilla* luciferase reporter for 24 h. MCF-7 cells and HT-1080 cells were preincubated with various concentrations of quinacrine for 1 h and then incubated with TNF-α (2.5 ng/mL) or without cytokines (–), in the presence or absence of quinacrine, for 2.5 h. Luciferase activity (fold) is shown as the mean ± SE of seven (**c**) and six (**d**) independent experiments. * *p* < 0.05, ** *p* < 0.01 and *** *p* < 0.001 indicate significant differences from the TNF-α stimulation without quinacrine. (**e**,**f**) MCF-7 cells (**e**) and HT-1080 cells (**f**) were preincubated with various concentrations of quinacrine for 1 h and then incubated with TNF-α (2.5 ng/mL) or without (control) cytokines, in the presence or absence of quinacrine, for 2.5 h. Cell viability was evaluated by the MTT assay. MTT reduction (%) is shown as the means ± SE of three independent experiments. * *p* < 0.05, ** *p* < 0.01 and *** *p* < 0.001 indicate significant differences from the control.

In order to support the results obtained with A549 cells, other cancer cell lines were tested. Human breast carcinoma MCF-7 cells were preincubated with quinacrine for 1 h and then incubated with TNF-α for 6 h. In MCF-7 cells, ICAM-1 expression augmented by the TNF-α stimulation was markedly reduced by quinacrine (Figure 4a), while cell viability was partially decreased by quinacrine under these conditions (Figure 4b). A549 cells and MCF-7 cells showed a similar sensitivity to quinacrine when incubated with quinacrine for 6 h, in the presence or absence of TNF-α (Figure 1b and Figure 4b). We also performed the NF-κB-dependent luciferase reporter assay using MCF-7 cells and human fibrosarcoma HT-1080 cells. MCF-7 and HT-1080 cells were preincubated with quinacrine for 1 h and then incubated with TNF-α for 2.5 h. Quinacrine at 50 µM inhibited TNF-α-induced NF-κB-dependent luciferase activity in MCF-7 cells with a slight reduction in cell viability (Figure 4c,e). Quinacrine also inhibited TNF-α-induced NF-κB-dependent luciferase activity in HT-1080 cells (Figure 4d), and a partial reduction was noted in cell viability (Figure 4f). Compared with A549 cells and MCF-7 cells, HT-1080 cells appeared to be more sensitive to quinacrine, when incubated with quinacrine for 2.5 h, in the presence or absence of TNF-α (Figure 2b and Figure 4e,f). These results confirmed that quinacrine inhibited TNF-α-induced ICAM-1 expression and NF-κB-dependent luciferase activity.

### 2.5. Quinacrine Does not Inhibit TNF-α-Induced IκBα Degradation

**Figure 5:**
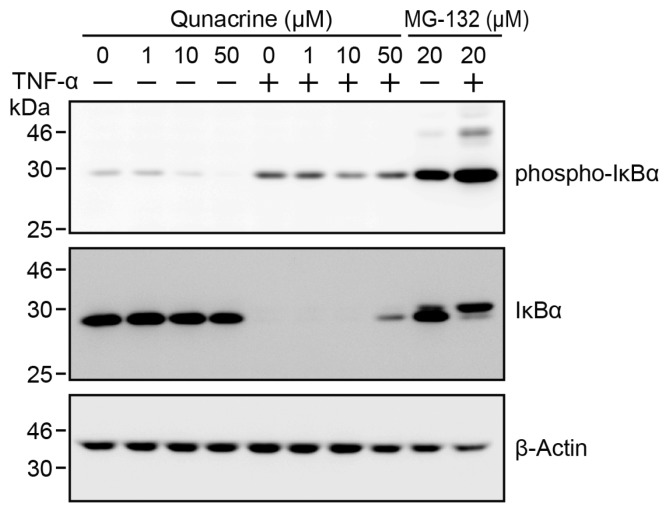
Quinacrine does not inhibit TNF-α-induced IκBα degradation. A549 cells were preincubated with various concentrations of quinacrine or MG-132 (20 µM) for 1 h and then incubated with (+) or without (−) TNF-α (2.5 ng/mL) for 15 min, in the presence or absence of quinacrine or MG-132. Western blotting was used to analyze cell lysates.

The IκB kinase complex is a common protein kinase, activated by TNF-α and IL-1α, and is essential for NF-κB activation [8,9,10]. We investigated the effects of quinacrine on TNF-α-induced IκBα phosphorylation and subsequent degradation. Our previous findings showed that IκBα phosphorylation was initiated within 5 min of TNF-α stimulation, and its subsequent degradation occurred within 15 min, in A549 cells [31]. A549 cells were preincubated with quinacrine for 1 h and then incubated with TNF-α for 15 min in the presence of quinacrine. The proteasome inhibitor MG-132 strongly inhibited IκBα degradation and thereby increased the phosphorylated form of IκBα in A549 cells (Figure 5). Quinacrine did not affect the amount of phospho-IκBα and only slightly decreased TNF-α-induced IκBα degradation at 50 µM (Figure 5). These results indicate that quinacrine only slightly, if at all, affects IκBα degradation in the NF-κB signaling pathway.

### 2.6. Quinacrine Does not Prevent TNF-α-Induced S-536 Phosphorylation or the Nuclear Translocation of p65

**Figure 6:**
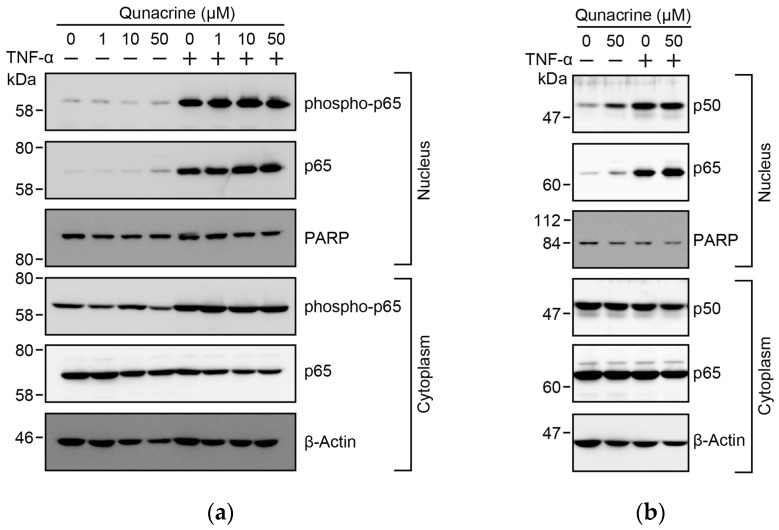
Quinacrine does not affect TNF-α-induced phosphorylation or the nuclear translocation of the NF-κB subunit p65. (**a**) A549 cells were preincubated, with or without various concentrations of quinacrine, for 1 h and then incubated with (+) or without (−) TNF-α (2.5 ng/mL) for 30 min, in the presence or absence of quinacrine. (**b**) A549 cells were preincubated, with or without quinacrine, for 1 h, and then incubated with (+) or without (−) TNF-α (2.5 ng/mL) for 30 min, in the presence or absence of quinacrine. Western blotting was used to analyze nuclear and cytoplasmic fractions.

Immediately after IκBα undergoes proteasomal degradation, the NF-κB heterodimers, p65 and p50, become free in the cytoplasm and translocate to the nucleus [8,10,11]. In addition, p65 is known to undergo phosphorylation at Ser-536 [4,14,15]. We previously reported that p65 was translocated to the nucleus from the cytoplasm, 30 min after a TNF-α stimulation, in A549 cells [31]. A549 cells were preincubated with quinacrine for 1 h and then incubated with TNF-α for 30 min. Quinacrine at 50 µM did not inhibit the nuclear translocation of p65 and p50 in A549 cells (Figure 6a,b). In A549 cells, TNF-α stimulation augmented the Ser-536 phosphorylation of p65 in the nucleus (Figure 6a). Quinacrine barely inhibited TNF-α-induced Ser-536 phosphorylation at concentrations up to 50 µM (Figure 6a). These results indicate that quinacrine does not prevent TNF-α-induced S-536 phosphorylation or the nuclear translocation of p65.

### 2.7. Quinacrine Inhibits the Binding of p65 to the ICAM-1 Promoter

**Figure 7:**
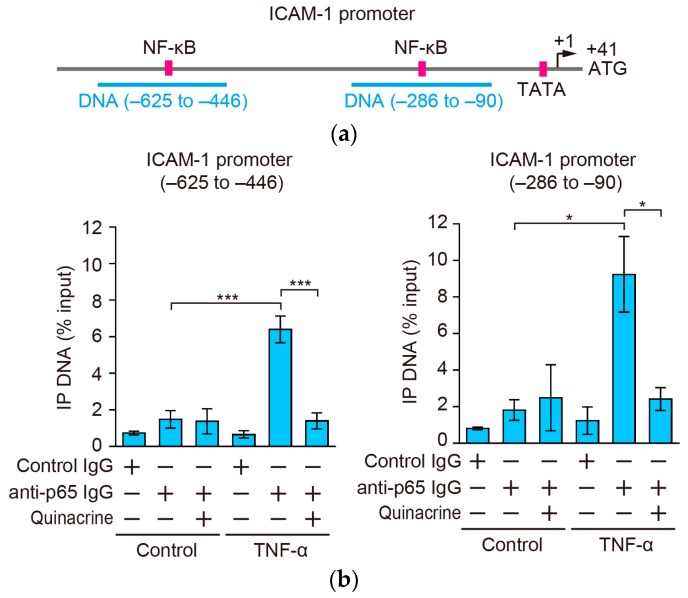
Quinacrine inhibits p65 binding to the ICAM-1 promoter. (**a**) The positions of κB sites [27,32], the TATA box, transcription start site (+1) [33,34], and translation start site (+41) are indicated. Two DNA sequences, amplified by quantitative PCR, are indicated; (**b**) A549 cells were preincubated, with (+) or without (−) quinacrine, for 1 h and then incubated, with or without TNF-α (2.5 ng/mL), for 30 min, in the presence or absence of quinacrine. Chromatin was immunoprecipitated with (+) or without (−) rabbit anti-p65 IgG or control rabbit IgG. The amounts of immunoprecipitated DNA were assessed by quantitative PCR. IP DNA (% input) is shown as the mean ± SE of three independent experiments. * *p* < 0.05 and *** *p* < 0.001 indicate significant differences from the control.

Two κB sites are located within 600 bp of the transcription start site [27,32] (Figure 7a). Hence, the direct binding of p65 to these κB sites of the ICAM-1 promoter in cells was analyzed by the chromatin immunoprecipitation (ChIP) assay. A549 cells were preincubated with quinacrine for 1 h and then incubated with quinacrine for 30 min. The TNF-α stimulation augmented the binding of p65 to the ICAM-1 promoter (Figure 7b). Quinacrine at 50 µM significantly inhibited TNF-α-induced p65 binding to the ICAM-1 promoter (Figure 7b). These results indicate that quinacrine inhibits the binding of p65 to the ICAM-1 promoter.

### 2.8. Quinacrine Sensitizes A549 Cells to TNF-α and the Fas Ligand

**Figure 8:**
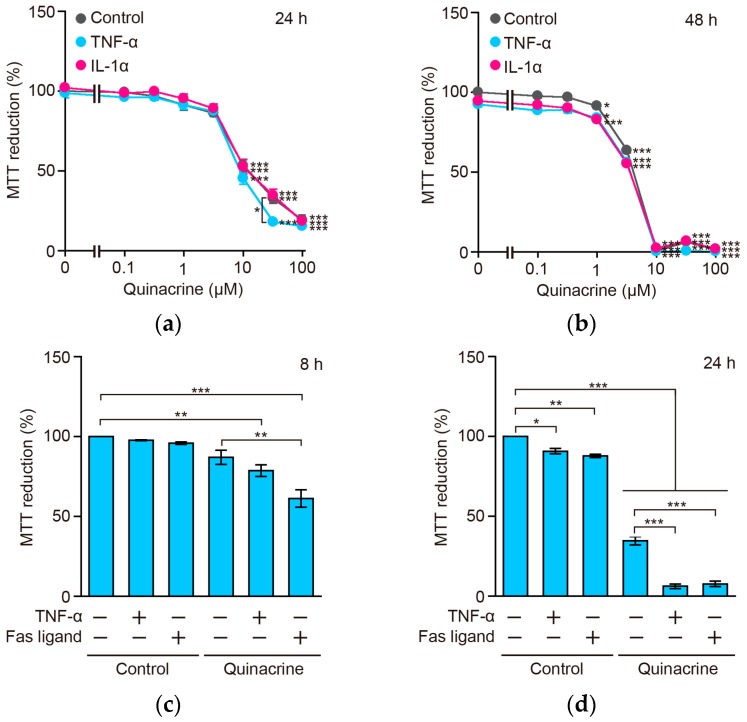

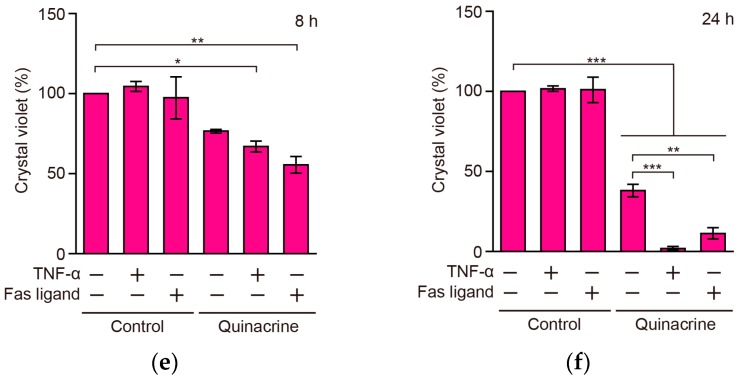
Quinacrine sensitizes A549 cells to TNF-α or the Fas ligand. (**a**,**b**) A549 cells were incubated with various concentrations of quinacrine, in the presence (+) or absence (−) of TNF-α (2.5 ng/mL) and IL-1α (0.25 ng/mL), for 24 h (**a**) and 48 h (**b**). Cell viability was measured by the MTT assay. MTT reduction (%) is shown as the mean ± SE of three independent experiments. Significant differences are shown by * *p* < 0.05 and *** *p* < 0.001; (**c**–**f**) A549 cells were incubated with (+) or without (−) quinacrine, in the presence (+) or absence (−) of TNF-α (100 ng/mL) and the FLAG-tagged Fas ligand (500 ng/mL) plus the anti-FLAG M2 antibody (500 ng/mL), for 8 h (**c**,**e**) or 24 h (**d**,**f**). Cell viability was evaluated by the MTT assay (**c**,**d**) and crystal violet staining (**e**,**f**); MTT reduction (%) and crystal violet (%) are shown as the mean ± SE of three independent experiments. * *p* < 0.05, ** *p* < 0.01 and *** *p* < 0.001 indicate significant differences.

The results showing that quinacrine inhibited TNF-α-induced NF-κB-dependent gene expression, prompted us to investigate whether quinacrine promoted TNF-α-induced cytotoxicity in A549 cells. A549 cells were incubated with TNF-α and IL-1α for 24 h and 48 h, in the presence or absence of quinacrine. The 3-(4,5-dimethylthiazol-2-yl)-2,5-diphenyltetrazolium bromide (MTT) assay revealed that quinacrine significantly reduced the viability of A549 cells at concentrations greater than 10 and 3.2 µM, for 24 and 48 h, respectively, irrespective of the treatment with TNF-α (2.5 ng/mL) or IL-1α (0.25 ng/mL) (Figure 8a,b). Nevertheless, it is important to note that cell viability was reduced significantly by TNF-α for 24 h in the presence of quinacrine at 32 µM (Figure 8a), suggesting that quinacrine sensitizes cells to TNF-α. In order to verify this, A549 cells were incubated with greater amounts of TNF-α (100 ng/mL) and shorter incubation times, in the presence of quinacrine. While TNF-α alone did not, or slightly affected, cell viability, TNF-α decreased cell viability almost completely in the presence of quinacrine for 24 h, and exerted a weaker effect for 8 h (Figure 8c,d). In contrast, the Fas ligand reduced cell viability more effectively during an 8-h incubation and almost completely during a 24-h incubation in the presence of quinacrine (Figure 8c,d). These results were further confirmed by crystal violet staining, which was used to measure adherent cells (Figure 8e,f). These results indicate that quinacrine has the ability to sensitize A549 cells to TNF-α and the Fas ligand.

### 2.9. Quinacrine Down-Regulates the Constitutive and TNF-α-Induced Expression of c-FLIP and Mcl-1

**Figure 9:**
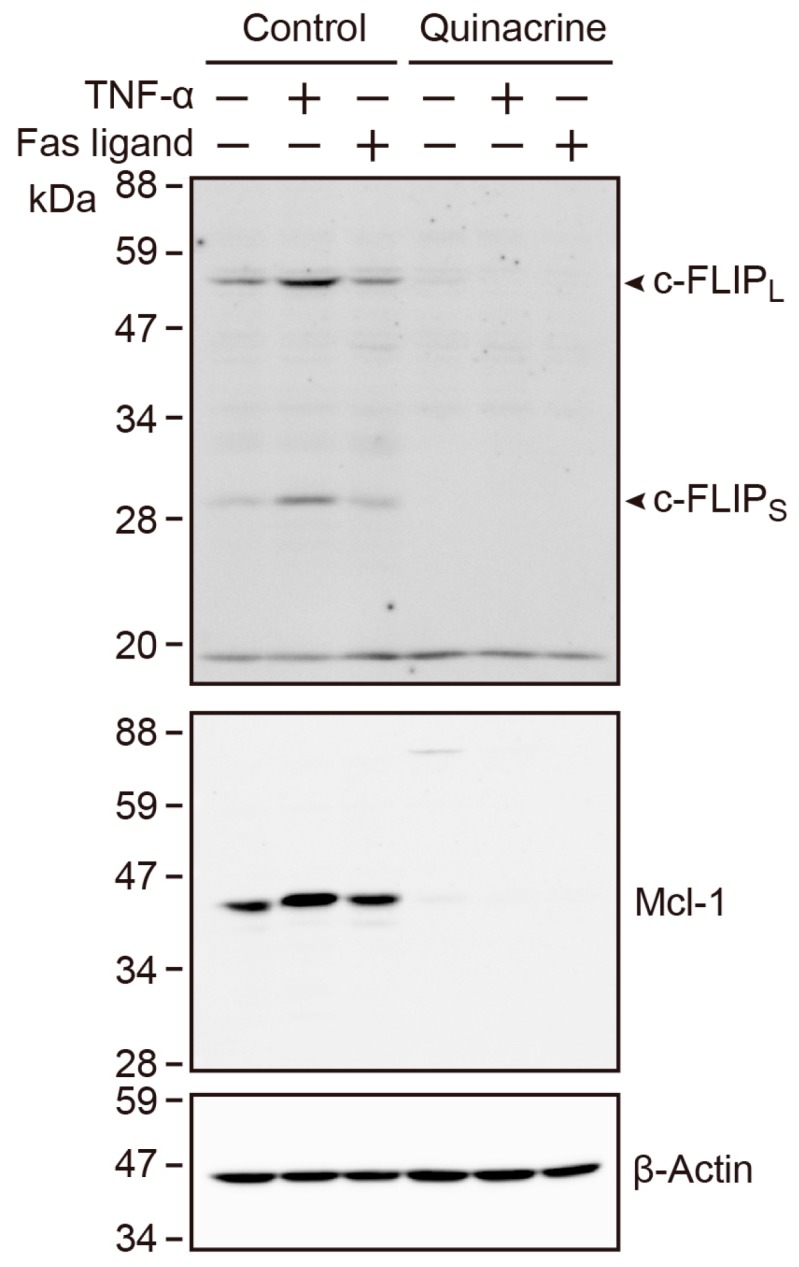
Quinacrine down-regulates the constitutive and TNF-α-induced expression of c-FLIP and Mcl-1. A549 cells were incubated with (+) or without (−) quinacrine, in the presence (+) or absence (−) of TNF-α (100 ng/mL) and the FLAG-tagged Fas ligand (500 ng/mL) plus the anti-FLAG M2 antibody (500 ng/mL), for 8 h. Western blotting was used to analyze cell lysates.

In order to gain an insight into the mechanisms underlying the sensitization of quinacrine to TNF-α and the Fas ligand, the expression of the anti-apoptotic proteins, cellular FLICE-inhibitory protein (c-FLIP) and Mcl-1, was investigated. A549 cells were incubated, with or without quinacrine, in the presence or absence of TNF-α or the Fas ligand for 8 h. A549 cells constitutively expressed c-FLIP_L_ (long form), c-FLIP_S_ (short form), and Mcl-1, and TNF-α up-regulated the expression levels of c-FLIP_L_, c-FLIP_S_, and Mcl-1 (Figure 9). Quinacrine diminished the constitutive and TNF-α-induced expression of c-FLIP_L_, c-FLIP_S_, and Mcl-1 (Figure 9).

## 3. Discussion

Quinacrine is widely used in the treatment of malaria and other diseases [16]. Its mechanisms of action in in vitro cell cultures are very complex, because it has been shown to possess multiple cellular targets [16]. In the present study, we found that quinacrine inhibited ICAM-1 expression and NF-κB-dependent luciferase activity, in response to TNF-α or IL-1α. Regarding the mechanisms of action of quinacrine on the NF-κB signaling pathway, we showed that quinacrine did not inhibit the nuclear translocation of p65 or its Ser-536 phosphorylation in A549 cells. In contrast, quinacrine strongly reduced the subsequent binding of p65 to the ICAM-1 promoter, indicating that quinacrine inhibits TNF-α-induced NF-κB-dependent transcriptional activation in A549 cells. Moreover, we found that quinacrine sensitized A549 cells to TNF-α or the Fas ligand. 

Previous studies reported that quinacrine inhibits the NF-κB signaling pathway and NF-κB-dependent gene expression [17,18,19,20,21,22,23,24]. Quinacrine has been reported to decrease the cellular levels of phospho-IκB kinase α/β, phospho-IκBα, and phospho-p65 at Ser-536 in human colon carcinoma cell lines [20], indicating that quinacrine blocks the NF-κB signaling pathway at a process upstream of IκB kinase activation [20]. Quinacrine either did not affect the Ser-536 phosphorylation of p65 in some colon cancer cell lines or reduced it in others [26]. On the other hand, 9-aminoacridine did not affect TNF-α-induced IκB degradation, but inhibited the Ser-536 phosphorylation of p65 [18]. Quinacrine has been shown to inhibit the step, downstream of p65 nuclear translocation and DNA binding in NF-κB-dependent transcription [21]. These previous findings demonstrated that quinacrine targets different steps on the NF-κB pathway in a cell type-specific manner. In the present study, we showed that quinacrine barely inhibited TNF-α-induced IκBα degradation, Ser-536 phosphorylation, or the nuclear translocation of p65 in A549 cells. These results indicate that quinacrine inhibits a process downstream of the nuclear translocation and phosphorylation of p65, at least in A549 cells.

Two consensus κB sites are located in the proximal region of the ICAM-1 promoter [27,32]. In the present study, the ChIP assay clearly showed that quinacrine inhibited the binding of p65 to the ICAM-1 promoter in intact A549 cells. The binding activity of p65 to the NF-κB consensus site was found to be markedly increased in nuclear extracts of H1299 cells treated with quinacrine [21]. This difference may be ascribed to different experimental settings, e.g., quinacrine concentrations and cell lines. As a potential mechanism, quinacrine may inhibit p65 binding to the ICAM-1 promoter by regulating p65 and its associated proteins. It has been reported that quinacrine has the ability to intercalate into DNA [16]. Another potential mechanism is that this biological activity of quinacrine may be responsible for the inhibition of p65 binding to the ICAM-1 promoter.

TNF receptor superfamily members trigger the NF-κB pathway and cell death pathway [35]. In the present study, we found that TNF-α or the Fas ligand reduced the viability of A549 cells more effectively in the presence of quinacrine. This is consistent with previous studies which have shown that quinacrine sensitizes human colon carcinoma and hepatocellular carcinoma to TRAIL [20,25]. We previously reported that A549 cells were resistant to the Fas ligand and TNF-α, and cycloheximide and cycloheximide plus GM6001 (a matrix metalloproteinase inhibitor added to prevent TNF receptor 1 ectodomain shedding) sensitized them to undergo caspase-8 activation, following a treatment with the Fas ligand and TNF-α, respectively [36]. In addition, the expression of the caspase-8 modulator c-FLIP_S_ was augmented by TNF-α, and down-regulated by cycloheximide in A549 cells [36]. In the present study, we found that quinacrine down-regulated the constitutive and TNF-α-induced expression of c-FLIP_L_, c-FLIP_S_, and Mcl-1 in A549 cells. Since c-FLIP is one of the NF-κB-responsive genes [37], it may be responsible for rendering resistance to TNF-α or the Fas ligand in A549 cells. Consistent with this, quinacrine has been reported to down-regulate the expression of c-FLIP_S_ and c-FLIP_L_ in human colon cancers [20]. Quinacrine also decreased the expression of Mcl-1, which was a target of NF-κB [20,25]. Since quinacrine inhibited NF-κB-dependent transcription, quinacrine appears to down-regulate the expression of NF-κB-responsive anti-apoptotic proteins, such as c-FLIP_L_, c-FLIP_S_, and Mcl-1, thereby promoting the activation of caspase-8 and cell death in A549 cells.

In conclusion, the present results revealed that quinacrine inhibits the NF-κB-dependent expression of ICAM-1 by preventing its binding to the ICAM-1 promoter. Moreover, quinacrine sensitized A549 cells to TNF-α or the Fas ligand. The NF-κB pathway plays an essential role in inflammation, immunity, and carcinogenesis [1,2,3,4,5]. Thus, the biological activities of quinacrine on the NF-κB pathway and cell death pathway may be important for therapeutics as anti-inflammatory and anti-cancer agents.

## 4. Materials and Methods

### 4.1. Cells

Human lung adenocarcinoma A549 cells (JCRB0076) and human fibrosarcoma HT-1080 cells (JCRB9113) were provided by the National Institutes of Biomedical Innovation, Health and Nutrition JCRB Cell Bank (Osaka, Japan). A549 cells, HT-1080 cells, and human breast carcinoma MCF-7 cells were maintained in RPMI 1640 medium (Thermo Fisher Scientific, Grand Island, NY, USA) supplemented with heat-inactivated fetal calf serum (Nichirei Biosciences, Tokyo, Japan) and penicillin-streptomycin mixed solution (Nacalai Tesque, Kyoto, Japan).

### 4.2. Reagents

Quinacrine was purchased from Sigma–Aldrich (St. Louis, MO, USA). Benzyloxycarbonyl-Leu-Leu-leucinal (MG-132) was obtained from the Peptide Institute (Osaka, Japan). Recombinant human TNF-α and IL-1α were kindly provided by Dainippon Pharmaceutical (Osaka, Japan). The FLAG-tagged human-soluble Fas ligand (139–281) was prepared, as described previously [38].

### 4.3. Antibodies

Primary antibodies reactive to β-actin (AC-15; Sigma–Aldrich, St. Louis, MO, USA), FLAG (M2; Sigma–Aldrich, St. Louis, MO, USA), ICAM-1 (15.2; Leinco Technologies, St. Louis, MO, USA), FLIP (Dave-2; Alexis^®^ Biochemicals, San Diego, CA, USA), IκBα (25; BD Biosciences, San Jose, CA, USA), Mcl-1 (D35A5; Cell Signaling Technology, Danvers, MA, USA), NF-κB p50 (H-119; Santa Cruz Biotechnology, Dallas, TX, USA), NF-κB p65 (C-20; Santa Cruz Biotechnology, Dallas, TX, USA), Poly(ADP-ribose) polymerase (PARP) (C2-10; Trevigen, Gaithersburg, MD, USA), phospho-IκBα (Ser32/36) (5A5; Cell Signaling Technology, Danvers, MA, USA), and phospho-NF-κB p65 (Ser536) (93H1; Cell Signaling Technology, Danvers, MA, USA) were obtained.

### 4.4. ICAM-1 Expression Assay

The amount of the cell-surface ICAM-1 protein was measured by cell-ELISA, as described previously [39,40]. ICAM-1 expression was calculated by measuring absorbance at 415 nm or 450 nm.

### 4.5. Cell Viability Assays

Cell viability assays, based on MTT and crystal violet, were performed, as described previously [30,39]. Cells were incubated with MTT at a final concentration of 500 µg/mL for the last 2 h of the incubation. Cells were solubilized with sodium dodecyl sulfate at a final concentration of 5% overnight. Absorbance at 570 nm was measured by an iMark^TM^ microplate reader (Bio-Rad Laboratories, Hercules, CA, USA). A549 cells were washed with PBS and stained with 0.2% crystal violet in methanol for 15 min, followed by an extensive wash with water. Absorbance at 570 nm was measured by iMark^TM^ microplate reader (Bio-Rad Laboratories, Hercules, CA, USA).

### 4.6. Assay for ICAM-1 mRNA Expression

Sepasol^®^-RNA I Super G (Nacalai Tesque, Kyoto, Japan) was used to extract total RNA. RNA (1 µg) was used to convert cDNA by ReverTra Ace^®^ (TOYOBO, Osaka, Japan). cDNA was used as a template for quantitative-PCR using SYBR^®^ Premix Ex Taq^TM^ II (Tli RNaseH Plus) (Takara Bio, Kusatsu, Japan) and the following primers: 5′-GCCTGGGAACAACCGGAAGGTG-3′ and 5′-GGGTGCCAGTTCCACCCGTTC-3′ for the 148-bp fragment of ICAM-1 [41] and 5′-GGACATCCGCAAAGACCTGTA-3′ and 5′-GCTCAGGAGGAGCAATGATCT-3′ for the 143-bp fragment of β-actin [42]. PCR was performed with Thermal Cycler Dice^®^ Real Time System Lite (Takara Bio, Kusatsu, Japan) under the following conditions: 94 °C for 3 min, followed by 45 cycles of 95 °C for 5 s, 58 °C for 30 s, and 72 °C for 30 s. The quantity of initial mRNA was calculated from primer-specific standard curves.

### 4.7. Luciferase Reporter Assay

Cells were transfected with the NF-κB-responsive firefly luciferase reporter plasmid (a kind gift from Ralph C. Budd) [43] and the cytomegalovirus promoter-driven *Renilla* luciferase reporter plasmid using the HilyMax transfection reagent (Dojindo Laboratories, Kumamoto, Japan). The preparation of cell lysates and the luciferase reporter assay were performed, as described previously [38]. Relative light units were immediately measured with Lumitester C-110 (Kikkoman Biochemifa, Tokyo, Japan).

### 4.8. Western Blotting

The preparation of cell lysates and Western blotting were performed according to previously described methods [39,40,44]. Cell lysates (30 µg) were separated by sodium dodecyl sulfate-polyacrylamide gel electrophoresis and transferred to nitrocellulose membranes, followed by an incubation with primary antibodies and horseradish peroxidase-conjugated secondary antibodies. Protein bands were visualized with ECL Western blotting detection reagents (GE Healthcare, Buckinghamshire, UK) or ImmunoStar^®^ Zeta (Wako Pure Chemical Industries, Osaka, Japan) and analyzed by ImageQuant LAS 4000 mini (GE Healthcare Japan, Tokyo, Japan).

### 4.9. ChIP Assay

A549 cells were fixed with 1% formaldehyde and chromatin was sheared by sonication. Immunoprecipitation was performed with Dynabeads^®^ protein A (Thermo Fisher Scientific, Vilnius, Lithuania) conjugated with 1 µg of a rabbit anti-p65 antibody (sc-372) or normal rabbit IgG (PP64; Millipore, Temecula, CA, USA) at 4 °C for 3 h. Immunoprecipitates were treated with ribonuclease A (250 µg/mL) at 50 °C for 1 h and then proteinase K (250 µg/mL) at 50 °C overnight, followed by purification using a Qiagen PCR purification kit (Qiagen, Hilden, Germany). Purified DNA was amplified with Thermal Cycler Dice^®^ Real Time System Lite (Takara Bio, Kusatsu, Japan) using SYBR^®^ Premix Ex Taq^TM^ II (Tli RNaseH Plus) (Takara Bio, Kusatsu, Japan). Primers were based on a previous study [45]: 5′-TCCCACGGTTAGCGGTCGCCG-3′ and 5′-CCTCTTTAATCGAGTGGATGAGCC-3′ for the ICAM-1 promoter (−625 to −446) and 5′-CGTGATTCAAGCTTAGCCTGG-3′ and 5′-CCTCCGGAACAAATGCTGCAG-3′ for the ICAM-1 promoter (−286 to −90). PCR conditions were: 94 °C for 3 min, followed by 45 cycles of 95 °C for 5 s, 58 °C for 30 s, and 72 °C for 30 s. The quantity of initial cDNA was calculated from primer-specific standard curves.

### 4.10. Statistical Analysis

The significance of differences was assessed by a one-way analysis of variance (ANOVA), followed by Tukey’s test for multiple comparisons. 

## Abbreviations

BSA	bovine serum albumin
c-FLIP	cellular FLICE-inhibitory protein
ChIP	chromatin immunoprecipitation
ICAM-1	intercellular adhesion molecule-1
IL-1	interleukin-1
HRP	horseradish peroxidase
IκB	inhibitor of nuclear factor κB
MTT	3-(4,5-dimethylthiazol-2-yl)-2,5-diphenyltetrazolium bromide
NF-κB	nuclear factor κB
PARP	poly(ADP-ribose) polymerase
PBS	phosphate-buffered saline
TNF	tumor necrosis factor
TRAIL	tumor necrosis factor-related apoptosis-inducing ligand
